# Predicting type 2 diabetes risk before and after solid organ transplantation using polygenic scores in a Danish cohort

**DOI:** 10.3389/fmolb.2023.1282412

**Published:** 2023-12-07

**Authors:** Quenia dos Santos, Preston Leung, Christian W. Thorball, Bruno Ledergerber, Jacques Fellay, Cameron R. MacPherson, Mads Hornum, Cynthia Terrones-Campos, Allan Rasmussen, Finn Gustafsson, Michael Perch, Søren S. Sørensen, Christina Ekenberg, Jens D. Lundgren, Bo Feldt‐Rasmussen, Joanne Reekie

**Affiliations:** ^1^ Centre of Excellence for Health, Institut Roche, Immunity and Infections (CHIP), Rigshospitalet, University of Copenhagen, Copenhagen, Denmark; ^2^ Precision Medicine Unit, Lausanne University Hospital and University of Lausanne, Lausanne, Switzerland; ^3^ School of Life Sciences, Ecole Polytechnique Fédérale de Lausanne, Lausanne, Switzerland; ^4^ Institut Roche, Boulogne-Billancourt, France; ^5^ Department of Nephrology, Copenhagen University Hospital Rigshospitalet, Copenhagen, Denmark; ^6^ Department of Surgical Gastroenterology, Rigshospitalet, Copenhagen, Denmark; ^7^ Department of Cardiology, Rigshospitalet, Copenhagen, Denmark; ^8^ Department of Clinical Medicine, Faculty of Health and Medical Sciences, University of Copenhagen, Copenhagen, Denmark

**Keywords:** type 2 diabetes mellitus, transplant, post-transplant diabetes mellitus, solid organ transplant recipient, polygenic risk score

## Abstract

Type 2 diabetes mellitus (T2DM) can be multifactorial where both genetics and environmental factors play a role. We aimed to investigate the use of polygenic risk scores (PRS) in the prediction of pre-transplant T2DM and post-transplant diabetes mellitus (PTDM) among solid organ transplant (SOT) patients. Using non-genetic risk scores alone; and the combination with PRS, separate logistic regression models were built and compared using receiver operator curves. Patients were assessed pre-transplant and in three post-transplant periods: 0–45, 46–365 and >365 days. A higher PRS was significantly associated with increased odds of pre-transplant T2DM. However, no improvement was observed for pre-transplant T2DM prediction when comparing PRS combined with non-genetic risk scores to using non-genetic risk scores alone. This was also true for predictions of PTDM in all three post-transplant periods. This study demonstrated that polygenic risk was only associated with the risk of T2DM among SOT recipients prior to transplant and not for PTDM. Combining PRS with a clinical model of non-genetic risk scores did not significantly improve the predictive ability, indicating its limited clinical utility in identifying patients at high risk for T2DM before transplantation, suggesting that non-genetic or different genetic factors may contribute to PTDM.

## Introduction

Post-transplant diabetes mellitus (PTDM) is a complication that can occur after a solid organ transplant (SOT). It refers to newly diagnosed diabetes mellitus following SOT, irrespective of diagnostic timing or whether T2DM was present but undetected before transplantation (Sharif et al., 2014). The prevalence of PTDM ranges from 10%–40% (Kasiske et al., 2003; Jenssen and Hartmann, 2019) and is associated with higher risk of death and complications after transplantation when compared to non-diabetes patients (Martínez-Dolz et al., 2005; Cho et al., 2012; Kim et al., 2017; Dos Santos et al., 2022).

Polygenic risk scores (PRS) are scoring profiles that extend information provided by genome wide association studies and focus on the collective contribution of individual genetic mutations to the phenotype of interest. While genome wide association studies provide *p*-values and effect sizes to assess the association between individual single nucleotide polymorphisms (SNPs) with the phenotype (Marees et al., 2018), these associations are treated as single events and those that have weak associations with the phenotype are often removed through multiple testing correction. PRS in contrast calculates a score to describe the effect of each SNP, thus it is possible to assess the risk for a given patient where multiple SNPs have been identified and can be treated collectively as a polygenic risk (Choi et al., 2020).

Previous studies assessing the use of PRS to predict T2DM have reported differing results. While some studies found PRS contributed very little to the prediction of T2DM in addition to a model with non-genetic risk scores for T2DM such as age, body mass index (BMI) and sex (Lango et al., 2008; Lyssenko et al., 2008; Chikowore et al., 2016), others demonstrated an improvement in the predictive performance of the model (Meigs et al., 2008; Chatterjee et al., 2013). As the knowledge in this area remains limited, this study aimed to assess the prediction of T2DM prior to transplant and PTDM among SOT recipients using non-genetic risk scores alone and the combination of non-genetic and PRS to investigate whether adding PRS could be useful for understanding the risk of T2DM and PTDM among SOT recipients.

## Patients and methods

### Study population

All patients ≥18 years old who underwent a SOT (heart, liver, lung, or kidney) at Rigshospitalet, Copenhagen University Hospital between January 2010 and December 2015 were eligible for inclusion. The study included SOT recipients who had an ethylenediaminetetraacetic acid stored blood sample in either the Region Hovedstadens Biobank (Rigshospitalet) or PERSIMUNE biobank (Rigshospitalet) and were part of the Management of post-Transplant infections in Collaborating Hospitals (MATCH) cohort (Lodding et al., 2015). For patients with more than one transplantation, only data related to the first transplant after 2010 were assessed**.**

### Data sources

Clinical characteristics, sociodemographic and biochemical data were extracted from the MATCH database and the Centre of Excellence for Personalized Medicine for Infectious Complications in Immune Deficiency (PERSIMUNE) data warehouse, (https://www.persimune.dk/), which includes both regional and nationwide data collected prospectively as part of routine care.

Information on prescribed medications including insulin and oral anti-diabetic medication were obtained from the Electronic Prescription Medication (EPM) database that had records of hospital prescriptions from 2006 to 2016, and the Danish Prescription Database, a database with outpatient prescription records from 2004 onwards (Dos Santos et al., 2022). There was a gap in the data from EPM from May 2011 to December 2011 due to a change in systems. Data on specific immunosuppressive therapies for individual patients were not available. However, detailed information on the immunosuppressive schemes per transplant type have been previously published by Ekenberg C et al. (Ekenberg et al., 2020).

Data on diagnoses were retrieved from the National Patient Registry (Lynge et al., 2011) and Sundhedsdatabanken. The National Patient Registry was established in 1977 and contains national data on all hospital admissions up to 2016 while Sundhedsdatabanken holds data records for patients in the capital region of Denmark from 2008 until 2016 (Dos Santos et al., 2022). Mortality data from the Danish Civil Registration System were used for death dates (Pedersen, 2011).

### Diabetes definition

Assessment of T2DM was consistent with a previously published study (Dos Santos et al., 2022). T2DM was assessed at pre-transplant and at three different PTDM time-periods.

1. 0–45 days post-transplant- “Early Likely PTDM” (EL-PTDM).
2. 46–365 days post-transplant
3. >365 days post-transplant

The fulfillment of at least one of the following criteria during the time-period of interest (for all time-periods, except before transplant), would classify the patients as “having developed diabetes mellitus”.

• A Hemoglobin A1C test ≥6.5 mmol/L or (Sharif et al., 2014).
• A prescription of antidiabetic medication from either EPM or Danish Prescription Database (use of insulin- Anatomical Therapeutic Chemical (ATC) code A10A, or use of oral antidiabetic medication -ATC code A10B) (Methodology WCCfDS, 2023).
• A diagnosis of diabetes (International Classification of Disease (ICD)-10 codes: E11, E13) (ICD10Data.com, 2023).

T2DM prior to transplant follows the above criteria with the exception of insulin treatment used during hospitalization (from EPM database) (Dos Santos et al., 2022), since these patients present a high incidence of corticoid-induced hyperglycaemia before transplant (Dos Santos et al., 2022). Additionally, prescription for antidiabetic medication during the first 15 days post-transplantation was not included in the definition of EL-PTDM due to a high prevalence of glucose intolerance and hyperglycaemia (Chakkera et al., 2009; Hecking et al., 2012). Patients classified with pre-transplant T2DM were classified as having T2DM in the entire follow-up period. Patients who were not classified as having pre-transplant T2DM were classified as PTDM if they met the T2DM definition in one of the time-periods of interest after-transplant, but could subsequently return to non-diabetes status in the following time-period if they did not meet the T2DM definition in the new time-period (Dos Santos et al., 2022).

SOT recipients with a diagnosis code for type-1 diabetes (E10) prior to transplantation were excluded as this study focuses on polygenic scores for T2DM.

### Genotyping, quality checks and imputation

Genotyping was performed using Infinium Global Screening Array-24 v1.0 BeadChip (Illumina). SNP array data were provided in plink file formats with an initial count of 673,642 SNPs and were lifted to GRCh37 using CrossMap (Zhao et al., 2014). Initial quality check was performed using PLINK software (v2.00a3LM) (Chang et al., 2015), filtering out individuals and SNPs with less than 90% genotyping and Hardy-Weinberg equilibrium *p*-value less than 1 × 10^−6^. Higher or lower than expected genotype heterozygosity was also performed through PLINK. Individuals with F-values more than three standard deviations above or below the F-value mean were removed from the data set. Subsequent quality checks such as strand flipping, position, frequency and reference/alternate allele checks were performed using PLINK through a helper script (HRC-1000G-check-bim-v4.3.0) developed by McCarthy Group tools (Centre for Human Genetics, 2023) with default recommended parameters while specifying the input population to be European. Reference files for this process used 1,000 Genomes Phase 3 combined data set (https://mathgen.stats.ox.ac.uk/impute/1000GP_Phase3.html) (Genomes Project et al., 2015). Phasing of the data was performed using SHAPEIT (v4.2) (Delaneau et al., 2019) and the imputation of SNPs was performed using IMPUTE5 (Rubinacci et al., 2020) using the 1,000 Genomes SNP set as reference panel and generated a total of 48,864,655 SNPs**.** Imputed SNPs with INFO score less than 0.8 were removed. An additional set of quality checks using the same parameters were performed on the imputed data to ensure high quality imputed SNPs. SNPs with minor allele frequency less than 1% were removed from the data set followed by an additional check on the heterozygosity count using same F-value filtering criterion. The final number of SNPs used for downstream analysis was 8,771,317.

### Genome-wide PRS

PRS selected for the MATCH cohort were obtained from a genome wide PRS study containing T2DM scores performed by Khera et al. (Khera et al., 2018) and downloaded from PGS Catalog (ID: PGS000014) (Lambert et al., 2021). This data set consisted of 6,917,436 SNPs generated from a European cohort. The scores for the MATCH cohort were calculated with PLINK (Chang et al., 2015) and a total of 3,134,520 SNPs were processed.

### Statistical analyses

Patient characteristics at the time of transplantation were described and compared for those with and without pre-transplant T2DM. Continuous variables were analysed using the Wilcoxon test (nonparametric data) and χ^2^ test was used for categorical variables.

PRS was split into quintiles for better visualization of their potentially non-linear associations with T2DM.

To construct the non-genetic risk score clinical risk factors were included in a multivariable logistic regression model predicting T2DM prior to transplant. The individual event probabilities were then used to create a ‘non-genetic score’ for each patient. The clinical risk factors were selected *a-priori* based on those previously identified in the literature (Lango et al., 2008; Lyssenko et al., 2008; Meigs et al., 2008), and available in our database. These included age at transplant (in years), sex, transplant type, BMI and Charlson Comorbidity Index (CCI) (per point) (Quan et al., 2005). For the CCI (Quan et al., 2005), the two dimensions related to T2DM (presence of T2DM with and without chronic complications) were excluded from calculation of the index to avoid collinearity issues with our outcome (Dos Santos et al., 2022). All non-genetic risk factors were calculated immediately prior to transplant and were not recalculated during follow-up.

This single measure of “non-genetic risk score” was then split into quintiles similar to the PRS. These non-genetic risk quintiles were then used to check and visualize interactions with the quintiles of the PRS. T2DM and PTDM event variation explained by the different univariable logistic regression models (non-genetic risk scores alone or PRS alone) and multivariable models (non-genetic risk scores + PRS) were documented with receiver operating characteristic values and the area under the curve (AUC) for each model was compared using χ^2^ tests. The area under the precision-recall curve (AUPRC) for each model is also presented. The quintiles for both the non-genetic risk scores and PRS were recalculated during each time-period to account for changes in the population. Sensitivity analyses including only patients that had a diagnosis code for T2DM prior to transplant and excluding those with a medication prescription and/or a haemoglobin A1C ≥ 6.5 mmol/L were performed to assess model consistency. For all PTDM analyses, patients with T2DM prior to transplantation were excluded.

An additional sensitivity analysis was performed, using Poisson regression analysis to account for the varying follow-up time of the SOT recipients. In this analysis patients were included from day 46 post-transplant and followed until the first time they met our definition of PTDM. Those not developing PTDM were censored at their date of death, new transplant date, or the end of follow-up (31.12.2016), whichever occurred first. All data analyses were performed using SAS 9.4.

## Results

A total of 959 SOT recipients had a transplant between January 2010 and December 2015. Due to the absence of available genetic information, 133 patients were excluded from this analysis. A further 121 patients with a diagnosis code for type-1 diabetes before transplant were also excluded. Thus, a total of 705 SOT recipients were included in the final data set.

Table 1 shows the patient characteristics prior to SOT. Prior to transplant, 521 patients were categorised as non-diabetes and 184 as having pre-transplant T2DM. Within the four SOT types, the percentage of patients with pre-transplant T2DM varied (*p* = 0.001). Heart transplant was the only transplant procedure to have more pre-transplant T2DM patients than non-diabetes patients (50.9% vs 49.1%). Additionally significant differences in the distributions were also identified between the groups when comparing BMI (*p* = 0.002) and age (*p* = 0.001). For BMI, 26.5% of the patients with BMI <25.0 had T2DM prior to transplant compared to 32.9% of the patients with BMI ≥25.0. In the age category, patients with pre-transplant T2DM were older (54.9 years, IQR: 46.7–62.1 years) than non-diabetic patients (49.4 years, IQR: 40.1–58.3 years).

**TABLE 1:** Characteristics of non-diabetes and diabetes patients at baseline.

Characteristics	Non-diabetes (N = 521)	Pre-transplant diabetes (N = 184)	*p*-value
**Type of Transplant - N (%)**			
Kidney	250 (71.0)	102 (29.0)	0.001
Liver	143 (86.7)	22 (13.3)	
Lung	99 (76.7)	30 (23.3)	
Heart	29 (49.1)	30 (50.9)	
**Sex- N (%)**			
Male	305 (73.0)	113 (27.0)	0.49
Female	216 (75.3)	71 (24.7)	
**BMI categories -N (%)**			
BMI<25.0	211 (73.5)	76 (26.5)	0.002
BMI≥25.0	151 (67.1)	74 (32.9)	
Missing	159 (82.4)	34 (17.6)	
**Age in years (Median and IQR)**	49.4 (40.1–58.3)	54.9 (46.7–62.1)	0.001
**CCI in points (Median and IQR)**	2.0 (2.0–3.0)	2.0 (1.0–3.0)	0.27

### Pre-transplant diabetes

**FIGURE 1:**
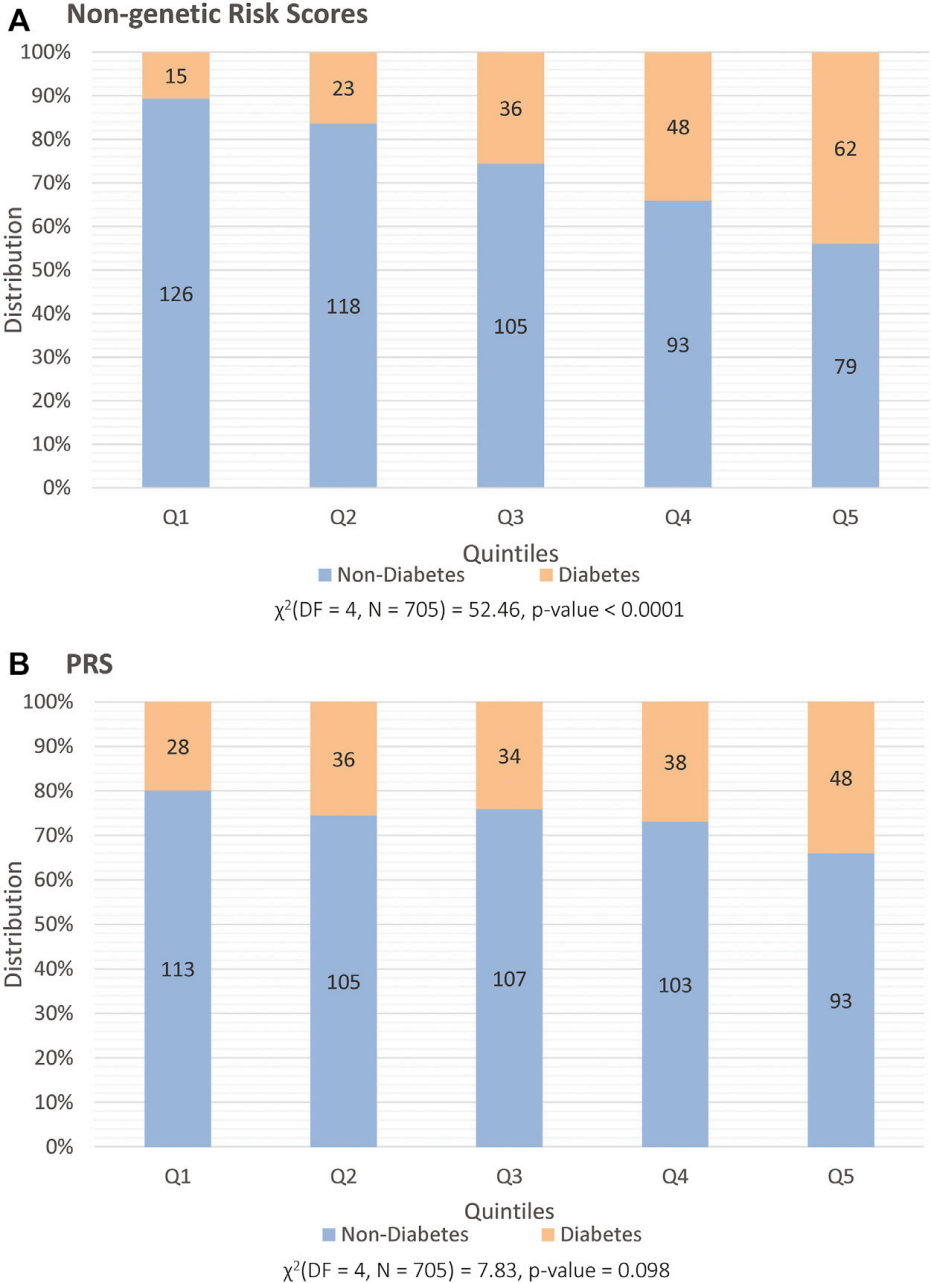
Distribution of patients prior to transplant, split into quintiles (x-axis) and categorized into those that are classified as having diabetes (orange) and non-diabetes (blue). The y-axis illustrates percentage distribution with each set of quintiles being based on **(A)** non-genetic risk factors and on **(B)** PRS.

The results of the multivariable logistic regression model used to generate the ‘non-genetic risk score’ are given in Supplementary Table S1 while results of the univariate logistic regression model are provided in Supplementary Table S2. Figures 1A,B show the distribution of pre-transplant T2DM and non-diabetes patients across the quintiles of both the ‘non-genetic risk score’ and the PRS respectively. There was a significant association with pre-transplant T2DM and the quintiles for the non-genetic risk score (*p* < 0.0001, Figure 1A), where a higher proportion with pre-transplant T2DM were found in the highest quintiles. This was also confirmed in the univariable logistic regression modelling in Table 2, where patients in the third quintile (Odds Ratio (OR): 2.87, 95% confidence interval (CI): 1.49–5.54), fourth (OR: 4.33, 95% CI: 2.28–8.20) and fifth quintiles (OR: 6.59, 95% CI: 3.50–12.37) had significantly higher odds of having T2DM than patients in the first quintile.

**TABLE 2:** Univariable logistic regression models with the non-genetic risk scores split into quintiles for the development of pre-transplant diabetes, EL-PTDM and PTDM in each study period.

Non-genetic risk scores	No. of patients	OR	95% CI	*p*-value	AUC	AUPRC	No. of events
**Pre-transplant**	705						184
1st quintile		1 (ref)	1 (ref)		0.67	0.26	15
2nd quintile		1.63	0.81–3.28	0.16			23
3rd quintile		2.87	1.49–5.54	0.001			36
4th quintile		4.33	2.28–8.20	<0.0001			48
5th quintile		6.59	3.50–12.37	<0.0001			62
**0–45 days post-transplant (EL-PTDM)**	521						60
1st quintile		1 (ref)	1 (ref)		0.61	0.11	11
2nd quintile		1.31	0.56–3.05	0.52			14
3rd quintile		0.51	0.18–1.45	0.21			6
4th quintile		0.89	0.36–2.21	0.81			10
5th quintile		1.86	0.84–4.15	0.12			19
**46–365 days post-transplant**	513						127
1st quintile		1 (ref)	1 (ref)		0.58	0.24	24
2nd quintile		1.00	0.52–1.90	1.00			24
3rd quintile		0.075	0.38–1.48	0.41			19
4th quintile		0.90	0.46–1.74	0.76			22
5th quintile		1.92	1.04–3.53	0.03			38
**>365 days post-transplant**	487						106
1st quintile		1 (ref)	1 (ref)		0.61	0.21	18
2nd quintile		0.98	0.47–2.03	0.97			18
3rd quintile		0.74	0.34–1.58	0.44			14
4th quintile		1.00	0.48–2.06	1.00			18
5th quintile		2.78	1.44–5.34	0.0022			38

The same trend was identified for the PRS, when assessing the proportion with pre-transplant T2DM across the quintiles (Figure 1B) despite a non-significant *p*-value (*p* = 0.09). Patients in the highest quintile of the PRS category were found to have higher odds of pre-transplant T2DM (OR: 2.08, 95% CI: 1.21–3.57, *p* = 0.007) when compared to those in the first quintile using univariable logistic regression (Table 3).

**TABLE 3:** Univariable logistic regression models with the PRS split into quintiles for the development of pre-transplant diabetes, EL-PTDM and PTDM in each study period.

PRS	No. of patients	OR	95% CI	*p*-value	AUC	AUPRC	No. of events
**Pre-transplant**	705						184
1st quintile		1 (ref)	1 (ref)		0.56	0.26	28
2nd quintile		1.38	0.79–2.42	0.25			36
3rd quintile		1.28	0.72–2.25	0.38			34
4th quintile		1.48	0.85–2.59	0.16			38
5th quintile		2.08	1.21–3.57	0.007			48
**0–45 days post-transplant (EL-PTDM)**	521						60
1st quintile		1 (ref)	1 (ref)		0.60	0.11	12
2nd quintile		0.39	0.13–1.15	0.08			5
3rd quintile		1.62	0.73–3.56	0.22			18
4th quintile		0.91	0.38–2.18	0.84			11
5th quintile		1.2	0.52–2.74	0.65			14
**46–365 days post-transplant**	513						127
1st quintile		1 (ref)	1 (ref)		0.53	0.24	25
2nd quintile		0.89	0.47–1.71	0.74			23
3rd quintile		1.23	0.66–2.31	0.49			29
4th quintile		0.84	0.44–1.62	0.61			22
5th quintile		1.18	0.63–2.20	0.60			28
**>365 days post-transplant**	487						106
1st quintile		1 (ref)	1 (ref)		0.55	0.21	18
2nd quintile		1.22	0.60–2.48	0.56			21
3rd quintile		1.44	0.72–2.86	0.29			24
4th quintile		0.94	0.45–1.96	0.87			17
5th quintile		1.62	0.82–3.21	0.16			26

**FIGURE 2:**
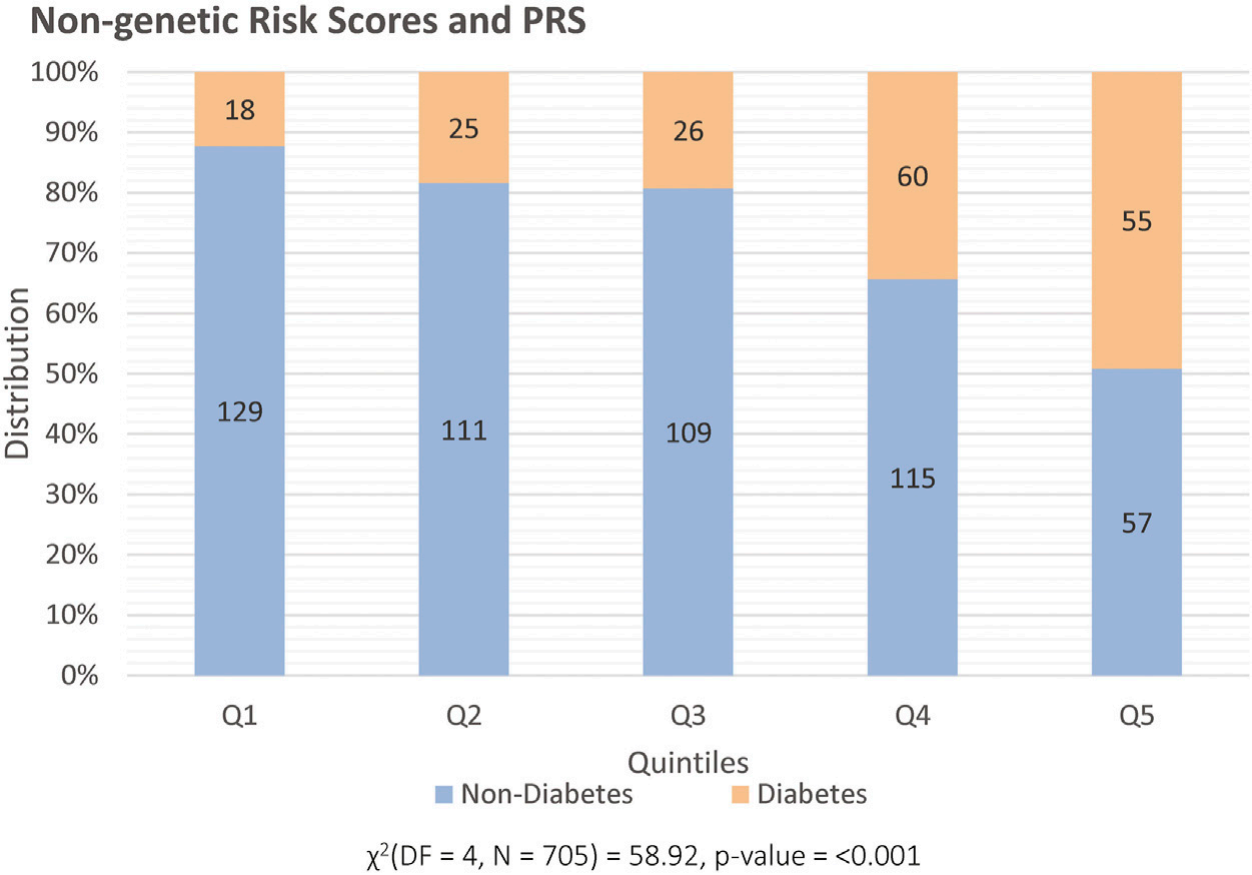
Distribution of patients prior to transplant, split into quintiles (x-axis) and categorized into those that are classified as having diabetes (orange) and non-diabetes (blue). The y-axis illustrates percentage distribution with each set of quintiles being based on the combined non-genetic risk factors and PRS.

Figure 2 illustrates the distribution of pre-transplant T2DM and non-diabetes patients in quintiles for the combined model of non-genetic risk factor and PRS, where a higher proportion of individuals with T2DM pre-transplant are observed in the highest quintile. Table 4 shows the AUC and the AUPRC for models including the non-genetic risk scores and models with both PRS and non-genetic risks scores for the prediction of pre-transplant T2DM. The addition of the PRS to the non-genetic risk scores model did not significantly improve the fit of the model (*p* = 0.10), although higher odds of pre-transplant T2DM were still observed among participants in the fifth quintile of PRS (adjusted OR: 1.94, 95% CI: 1.10–3.40, *p* = 0.02) when compared to the first quintile. Sensitivity analysis only defining T2DM using an ICD10 diagnosis code for T2DM prior transplant showed consistent results (Supplementary Table S3).

**TABLE 4:** Area under the curve (AUC) for multivariable models including the non-genetic risk scores^*^, and combined PRS and non-genetic risks factors for the prediction of pre-transplant diabetes, EL-PTDM and PTDM.

Time period	AUC	AUPRC	*p-value* ^a^
**Pre-transplant diabetes**			
Model including non-genetic risk scores alone	0.67	0.26	0.10
Model including non-genetic risk scores + PRS	0.68	0.26	
**0–45 days post-transplant**			
Model including non-genetic risk scores alone	0.61	0.11	0.09
Model including non-genetic risk scores + PRS	0.65	0.11	
**46–365 days post-transplant**			
Model including non-genetic risk scores alone	0.58	0.24	0.25
Model including non-genetic risk scores + PRS	0.60	0.24	
**>365 days post-transplant**			
Model including non-genetic risk scores alone	0.61	0.21	0.53
Model including non-genetic risk scores + PRS	0.62	0.21	

a - *p*-values generated using χ^2^ test comparing AUC.

* - Multivariable models included the PRS, and the non-genetic risk score, both stratified into quintiles and fitted as categorical variables. Quintiles were recalculated for each time-period. The logistic regression model used to generate the non-genetic risk score is given in Supplementary Table S1

### Post-transplant diabetes (PTDM)

At 0–45 days after transplant, 60 (11.5%) of the 521 non-diabetes patients had EL-PTDM.

For the period of 46–365 days post-transplant, a total of eight patients were excluded from the analysis (four died and four censored at the end of follow-up). None of the eight were classified as EL-PTDM. This resulted in a total of 513 patients being assessed in the 46–365 days post-transplant period. A total of 127 (24.8%) patients were defined as having PTDM in this period (48 that also had EL-PTDM, and 79 newly diagnosed).

In the last period (>365 days post-transplant), a further 26 patients were excluded from the analysis (25 who had died in the year following transplant and one who had less than a year’s follow-up). Thirteen of the 26 patients excluded had PTDM in the earlier period. Thus 487 patients were assessed for PDTM >365 days after transplant of which 106 (21.8%) met our definition of PTDM (65 who also had PTDM in the early period and 41 newly classified).

During the 46–365 and >365 days post-transplant period, the highest quintile of the non-genetic risk scores was associated with an increased odds of PTDM (OR: 1.92, 95% CI: 1.04–3.53, *p* = 0.03, and OR: 2.78, 95% CI: 1.44–5.34, *p* = 0.002, respectively) compared to the lowest quintile. An increased odds of EL-PTDM (OR: 1.86, 95% CI: 0.84–4.15, *p* = 0.12) was also observed in the highest quintile however this difference was not statistically significant (Table 2). No significant associations were observed between the odds of developing EL-PTDM or PTDM and the PRS, in any of the time-periods in univariable analysis (Table 3).

Comparison between the AUC of the non-genetic risk scores model and the AUC with the addition of the PRS is shown in Table 4. Overall, the AUC was lower for all the models in the post-transplant periods when compared to the models for pre-transplant T2DM. Additionally, no significant improvement in the AUC was observed in any of the time-periods for predicting EL-PTDM or PTDM when the PRS was added to the model.

### Sensitivity analysis using Poisson regression

513 patients were included in the sensitivity analysis using Poisson regression analysis to account for the different follow-up times. Individuals in this analysis were included from day 46-post transplant and followed until the first time a patient met our definition for PTDM, at a minimum of 46 days after transplant. Patients in the fifth quintile of non-genetic risk score had higher rate of PTDM (incidence rate ratio (IRR) 1.68, 95% CI: 0.98–2.89, *p* = 0.05) during follow-up (from day 46 post-transplant), compared to patients in the first quintile. However, similar to the main analysis, there was no significant association identified between the PRS and PTDM when it was included in the model with the non-genetic risk score (global *p*-value = 0.23). However, patients in the fifth quintile of PRS were observed to have a two times higher rate of PTDM (adjusted IRR 2.00, 95% CI: 1.07–3.75, *p* = 0.02) when compared to patients in the first quintile (see Supplementary Table S4).

## Discussion

The aim of this study was to assess whether a PRS for T2DM was associated with pre-transplant T2DM and whether the additional information from a PRS could improve the prediction of PTDM among SOT recipients compared to one with non-genetic scores alone. While we found that a high PRS was associated with increased odds of pre-transplant T2DM, including the PRS in a model with non-genetic risk scores, did not significantly improve prediction of T2DM compared to the model with non-genetic risk scores alone. Further, when considering EL-PTDM and PTDM in various time-periods, neither the non-genetic risk scores nor the PRS were strongly associated with the outcome.

For the pre-transplant period, patients in the highest quintile of the PRS and patients in the third, fourth and fifth quintiles of the non-genetic risk were found to have had higher odds of having T2DM. The addition of the PRS did not significantly improve the model compared to the non-genetic information alone. Sensitivity analysis including only patients with ICD-10 diagnosis codes for T2DM prior to transplant also had similar results. Our results are consistent with some of the previous studies assessing PRS to predict T2DM which also did not show a significant improvement of T2DM prediction when the PRS was added to a model with non-genetic risk scores (Lango et al., 2008; Lyssenko et al., 2008). A systematic review including studies that assessed PRS to predict T2DM (Padilla-Martínez et al., 2020) through comparing AUC of different models with and/or without the genetic information showed in most of the cases, the addition of the PRS to the non-genetic risk scores had a modest effect on the ability to predict T2DM (Lango et al., 2008; Lyssenko et al., 2008; Chikowore et al., 2016) at the best case scenario.

Some studies recorded better results when incorporating the genetic information with the clinical risk factors, but this improvement on T2DM prediction was small. While this could be due to the small number of SNPs when generating the PRS, it also suggests that clinical risk factors play a larger role than genetic factors alone. For example, in a study conducted by Meigs et al., (Meigs et al., 2008), using 18 SNP PRS, the AUC for T2DM prediction adjusted for age, sex and family history overall showed minor improvements. In our study, over six million SNPs from a PRS generated to predict T2DM (Lambert et al., 2021) were mapped to over three million SNPs of the MATCH cohort. Yet similarly, minor improvements were observed on only a certain portion of the data. Thus, our findings further extend the idea that PRS has a small effect when used in addition to non-genetic factors in the prediction of T2DM.

PTDM was considered at three different periods, EL-PTDM which was assessed from day 0 to day 45 post-transplant, then PTDM assessed at both 46–365 days post-transplant with the last period at >365 days post-transplant. No significant association was observed between PRS and a diagnosis of PTDM in any of the time-periods considered. Shaked et al. performed the only study which examined PTDM and PRS in liver and kidney recipients. Their study demonstrated that recipient T2DM PRS were independently associated with PTDM risk between 6 and 12 months after transplantation in both liver and kidney transplant recipients. This significantly improved PTDM prediction compared with a model that included only non-genetic risk scores for PTDM (Shaked et al., 2022). They also reported that T2DM PRS in liver donors, but not in kidney donors, was an independent risk factor for PTDM development. In our cohort, we did not have genetic information for the donors, thus it was not possible to generate a PRS for them and assess if the addition of this information would improve the performance of the model in terms of PTDM prediction. Furthermore, we had insufficient power to consider the transplant types individually.

Interestingly, the clinical model alone did not appear to be as good at predicting EL-PTDM or PTDM when compared to predicting pre-transplant T2DM. Individuals who met our definition of PTDM, particularly in the earlier periods post-transplant may contain a mixture of those who were already at an increased risk for T2DM and those where it was a consequence of the transplant regimen. As we only considered clinical factors measured prior to transplant there may be other treatment related clinical factors such as immunosuppressive regimen including steroid use that are more important for the development of PTDM following a SOT. Additionally, it is important to note this may also be a potential explanation for the lack of association between the PRS and PTDM, as individuals with a high genetic risk for T2DM may have already been diagnosed prior to their transplant and thus were excluded from this part of our analysis. After the removal of the group with T2DM prior to transplant, the interplay between genetic risk, and clinical risk factors following transplant maybe very different.

The limitations of this study should be highlighted. Firstly, this study did not include patient self-report of diabetes status but relied on a definition to identify T2DM patients used in a previous study (Dos Santos et al., 2022). However, several studies (Miller et al., 2004; Dos Santos et al., 2022) have adopted definitions to identify patients with T2DM based on similar criteria and they all performed quite well. Additionally, validation can also be derived from our finding that the highest quintile of the PRS score was significantly associated with pre-transplant T2DM. Secondly, the risk scores used in this study was generated based on a PRS for T2DM in a general European population. This is different to post-transplant patients where some other factors, such as stress from surgery and medication, can have an impact in the genetic information (Shaked et al., 2022). One additional limitation is the lack of information about the immunosuppressive medication. It is well known that immunosuppressive medication has great influence on glucose metabolism (Boloori et al., 2015), so this would be an important adjustment variable for our analyses if it had been available. Additionally, our results were based on a cohort of SOT recipients from one hospital in Denmark, validation in an independent cohort would add strength to the generalisability of our findings.

Strengths of this study are also worth mentioning. This was the first study that proposed the use of T2DM PRS to predict PTDM in a large and more varied cohort, including heart, liver, kidney, and lung recipients and in different time-periods after transplant (0–45 days after transplant, 46–365 days and >365 days after transplant). To our knowledge, there is only one study assessing the use of T2DM polygenic score to predict PTDM in liver and kidney recipients at 6–12 months after transplant (Shaked et al., 2022).

In summary, this study demonstrated that among SOT recipients, PRS could, help in the identification of patients at risk for T2DM prior to transplant. However, traditional non-genetic risk scores were just as good at predicting T2DM prior to transplant and the PRS did not provide any improvement in identification of high-risk individuals indicating its limited potential clinical utility Furthermore, the same predictive ability of PRS was not observed for PTDM, suggesting that non-genetic or different genetic factors, possibly related to the transplant itself, may contribute to the development of PTDM. Future studies assessing prediction of PTDM with the use of polygenic scores for T2DM are needed.

## Data Availability

The data analyzed in this study is subject to the following licenses/restrictions: Data requests must be reasonable and approved by PERSIMUNE Scientific Committee prior to data sharing. Requests to access these datasets should be directed to persimune.rigshospitalet@regionh.dk.

## Abbreviations

Area under the curve (AUC), area under the precision-recall curve (AUPRC), body mass index (BMI), Charlson Comorbidity Index (CCI), confidence interval (CI), Early Likely PTDM (EL-PTDM), Electronic Prescription Medication (EPM), Management of post-Transplant infections in Collaborating Hospitals (MATCH), odds ratio (OR), polygenic risk scores (PRS), post-transplant type 2 diabetes mellitus (PTDM), single nucleotide polymorphisms (SNPs), solid organ transplant (SOT), Type 2 diabetes mellitus (T2DM).
